# Clinical analysis of adult-onset spinocerebellar ataxias in Thailand

**DOI:** 10.1186/1471-2377-14-75

**Published:** 2014-04-05

**Authors:** Pairoj Boonkongchuen, Sunsanee Pongpakdee, Panitha Jindahra, Chutima Papsing, Powpong Peerapatmongkol, Suppachok Wetchaphanphesat, Supachai Paiboonpol, Charungthai Dejthevaporn, Surat Tanprawate, Angkana Nudsasarn, Chanchai Jariengprasert, Dittapol Muntham, Atiporn Ingsathit, Teeratorn Pulkes

**Affiliations:** 1 Department of Medicine, Division of Neurology, Faculty of Medicine, Ramathibodi Hospital, Mahidol University, Bangkok, Thailand; 2 Division of Medicine, Bhumibol Adulyadej Hospital, Bangkok, Thailand; 3 Department of Medicine, Division of Neurology, Buriram Hospital, Buriram, Thailand; 4 Department of Medicine, Division of Neurology, Ratchaburi Hospital, Ratchaburi, Thailand; 5 Department of Internal Medicine, The Northern Neuroscience Center and Division of Neurology, Faculty of Medicine, Chiang Mai University, Chiang Mai, Thailand; 6 Department of Otolaryngology, Division of Otoneurology, Faculty of Medicine, Ramathibodi Hospital, Mahidol University, Bangkok, Thailand; 7 Section for Clinical Epidemiology and Biostatistics, Faculty of Medicine, Ramathibodi Hospital, Mahidol University, Bangkok, Thailand

**Keywords:** Autosomal dominant cerebellar ataxia, Spinocerebellar ataxia, SCA, Saccade, Ophthalmoplegia

## Abstract

**Background:**

Non-ataxic symptoms of spinocerebellar ataxias (SCAs) vary widely and often overlap with various types of SCAs. Duration and severity of the disease and genetic background may play a role in such phenotypic diversity. We conducted the study in order to study clinical characteristics of common SCAs in Thailand and the factors that may influence their phenotypes.

**Methods:**

131 (49.43%) out of 265 Thai ataxia families with cerebellar degeneration had positive tests for SCA1, SCA2, Machado-Joseph disease (MJD) or SCA6. The study evaluated 83 available families including SCA1 (21 patients), SCA2 (15), MJD (39) and SCA6 (8). Comparisons of frequency of each non-ataxic sign among different SCA subtypes were analysed. Multivariate logistic regression analyses were undertaken to analyze parameters in association with disease severity and size of CAG repeat.

**Results:**

Mean ages at onset were not different among patients with different SCAs (40.31 ± 11.33 years, mean ± SD). Surprisingly, SCA6 patients often had age at onset and phenotypes indistinguishable from SCA1, SCA2 and MJD. Frequencies of ophthalmoparesis, nystagmus, hyperreflexia and areflexia were significantly different among the common SCAs, whilst frequency of slow saccade was not. In contrast to Caucasian patients, parkinsonism, dystonia, dementia, and facial fasciculation were uncommon in Thai patients. Multivariate logistic regression analysis demonstrated that ophthalmoparesis (*p* < 0.001) and sensory impairment (*p* = 0.025) were associated with the severity of the disease.

**Conclusions:**

We described clinical characteristics of the 4 most common SCAs in Thailand accounting for almost 90% of familial spinocerebellar ataxias. There were some different observations compared to Caucasian patients including earlier age at onset of SCA6 and the paucity of extrapyramidal features, cognitive impairment and facial fasciculation. Severity of the disease, size of the pathological CAG repeat allele, genetic background and somatic heterogeneity of pathological alleles may influence clinical expressions of these common SCAs.

## Background

Spinocerebellar ataxias (SCAs) are genetically heterogeneous neurodegenerative disorders associated with progressive ataxia or less commonly episodic ataxia. Adult-onset patients mainly inherit the condition by autosomal dominant transmission. Phenotypes often accompany various non-ataxic signs including saccadic abnormalities, ophthalmoplegia, extrapyramidal features, pyramidal signs, polyneuropathy, amyotrophy, fasciculation, dementia, and macular degeneration [1]. To date, the Human Genome Nomenclature Committee has assigned types of SCAs into SCA1 to SCA37 in association with 31 chromosomal loci and genes [1-5]. Machado-Joseph disease (MJD) or SCA3 is generally the most common form of SCA worldwide [6-8]. Other common types of SCAs in various ethnic groups include SCA1, SCA2, SCA6, SCA7 and dentatorubro-pallidoluysian atrophy (DRPLA). All these common SCAs are caused by expansions of polyglutamine-encoding CAG repeat [1].

Phenotypes of different SCAs often overlap and are indistinguishable resulting in a difficulty in making clinical diagnosis. However, some clinical clues and epidemiological data of common SCAs in each population may be helpful for setting up appropriate diagnostic genetic tests [9,10]. For example, pyramidal signs are more common in SCA1 and SCA3 while peripheral neuropathy is more common in SCA2 [9,11,12]. Slow saccades may be more prevalent in SCA2 [13,14]. Sizes of expanded repeat allele, age at onset, age, and disease duration have been described as factors that might influence clinical expression of the common SCAs [15-17]. Although neuropathology of the polyglutamine-associated SCAs is generally widespread over the cerebellum, brainstem and cerebral hemisphere in the advance stage of the diseases [18], observations of some non-ataxic signs such as slow saccade in presymptomatic carriers or at the early symptomatic stage suggest that other neurons apart from cerebellar neurons may also be similarly vulnerable to the pathological process caused by the polyglutamine-associated SCAs [19,20]. Slow saccade represents loss of excitatory burst neuronal function, whose neurons initiate the pulse sequence of saccadic eye movement [21]. Therefore degenerative process of those neurons in brainstem should take place at a very early stage of the disease in those patients [19,22]. Although slow saccades in SCA2 patients may be influenced by the size of polyglutamine tract [17], this mechanism cannot solely explain the presence or absence of slow saccades in all SCA2 patients. Other genetic factors may also play a role in selective neuronal susceptibility and phenotypic expression of the polyglutamine-associated SCAs [23].

MJD is the most common SCA in the Thai population followed by SCA1, SCA2 and SCA6 [24]. Over the past 15 years, the study group has had experience of seeing over a hundred Thai SCA families. Interestingly, we have hardly observed some of the frequently described non-ataxic signs comprising facial fasciculation, parkinsonism, dystonia, and dementia in those patients. This finding implies that those signs might be less common compared to previous reports of other ethnic groups. Furthermore, slow saccade was commonly observed in all subtypes of the common SCAs. Therefore the study was conducted in order to analyze the clinical characteristics of the Thai patients with adult-onset SCAs and identify factors that may influence the clinical expression of SCAs. Since we have observed a clinical concordance in the patients from the same family, we thus recruited only the index patient from each family into the study.

## Methods

### Patients

We reviewed medical records of all patients with progressive cerebellar ataxia, who were tested for SCA1, SCA2, MJD, SCA6, SCA7 and DRPLA at the Division of Neurology, Department of Medicine, Ramathibodi Hospital. After undertaking intensive investigations, some of the patients were identified by other diagnoses including cerebellar variant of multiple system atrophy, neurogenic weakness, ataxia and retinitis pigmentosa (NARP) and autoimmune cerebellar disorders. The remaining patients were from 265 families. 131 families (49.43%) tested positive for SCA1 (38 families), SCA2 (22), MJD (61) and SCA6 (10). 75 of those families (57.25%) had family history of ataxia. The study identified SCA1, SCA2, MJD, or SCA6 mutations in almost 90% of the patients in the familial group (75 of 86 families), while only 31% of the sporadic cases (56 of 179 cases) were identified as one of these common SCAs (Table 1). We have not identified patients with SCA7 and DRPLA so far. Details of the frequencies of SCAs were listed in Table 1.

**Table 1:** Frequencies of the common spinocerebellar ataxias in Thai patients

	**Familial group (%) N = 86**	**Sporadic group (%) N = 179**	**Total (%) n = 265**
SCA1	19 (22.10)	19 (10.61)	38 (14.34)
SCA2	10 (11.63)	12 (6.70)	22 (8.30)
MJD	40 (46.51)	21 (11.73)	61 (23.02)
SCA6	6 (6.98)	4 (2.23)	10 (3.77)
Total	75 (87.21)	56 (31.28)	131 (49.43)
Patients without mutation	11 (12.79)	123 (68.72)	134 (50.57)

*Abbreviations as follows:SCA* spinocerebellar ataxia, *MJD* Machado-Joseph disease.

The study subsequently enrolled 83 index patients with SCA1, SCA2, MJD and SCA6 from the database, who were available for clinical review and examination. All accessible family members were also evaluated. However, statistical analyses only used data of the index cases. The examination processes were undertaken at 4 participating hospitals during April 2010 to June 2013. All of the participants were evaluated by T.P. and P.B., or S.Po., or P.J. except for 2 families, who live in rural areas of Phayao province in the northern part of Thailand. They were examined by P.J. and S.T. Collected patient’s profiles and clinical data included sex, the family’s hometown, age at onset, disease duration, age at diagnosis, family pedigrees, cerebellar signs, fundoscopic examination, eye movement abnormalities, nystagmus, muscle tone, tendon reflexes, Babinski’s sign, parkinsonism, dystonia, chorea, dementia (Mini-mental state examination), fasciculation, sensory examination (vibration, joint position and pin prick sensations) and scale for the assessment and rating of ataxia (SARA). All patients provided both verbal and written informed consent. The research protocol was approved by the ethics committee or hospital ethical review board of every participating university and hospital.

### Genetic analysis

DNA samples were extracted from peripheral leukocyte by phenol-chloroform method or using QIAGEN DNA purification kit (QIAGEN, CA, USA). Fluorescently-labelled PCR of the expanded repeat alleles of the *ATXN1* (ataxin 1)*, ATXN2, ATXN3, CACNA1A* (alpha 1A subunit, P/Q type voltage-dependent calcium channel: SCA6)*, ATXN7* and *ATN1* (atrophin 1: DRPLA) genes were performed by using primer sets as previously described methods [9,11,25-28]. The allele sizes were then determined by running the PCR products on Beckman CEQ8800 DNA Analysis System (Beckman Coulter, CA, USA). Sizes of CAG repeat of both normal and pathological alleles of each chromosome were calculated by comparing results with the size of a normal control sample, of which the sizes were prior defined by direct sequencing. In order to avoid a false negative result caused by a very large expanded allele failing to be amplified by using regular PCR [29]. all samples whose results showed patterns of homozygous wild-type alleles of *ATXN1, ATXN2* and *ATXN3*, were subsequently re-analyzed by performing long-range PCR using LA Taq polymerase (TaKaRa, Chiba, Japan).

### Statistical analysis

Results were expressed as means ± standard deviations. One-way analysis of variance (ANOVA) was used to compare means of each continuous variable among different SCAs. Categorical variables were compared by using chi squared test. Test results considered significant differences at the level of p-value < 0.05. Comparisons of clinical characteristics between groups of patients with SARA score <15 and ≥15 were determined by logistic regression analysis. The results were expressed as odd ratio (OR), 95% confidence intervals (CI) and p-values. After a number of univariate predictive factors had been determined, forward stepwise selection was carried out to determine the appropriate multivariate model. Factors selected for the multivariate model were those found significant in the univariate model. Results were considered statistically significant for p <0.10 of univariate and p <0.05 of multivariate analysis. Whether size of the pathological SCA3 repeat allele influences the clinical outcomes was tested by logistic regression analysis and then adjusted with other factors including age at onset, age, sex and duration of the disease. All analyses were performed using STATA version 12 (Stata Corp., College Station, TX, USA).

## Results

### Patient characteristics

MJD is the most common type of adult-onset spinocerebellar ataxias in the study followed by SCA1, SCA2 and SCA6, respectively (Table 1). Eighty-three index patients were enrolled on the study including 39 MJD patients, 21 SCA1, 15 SCA2 and 8 SCA6 patients. Analyses of their family pedigrees identified at least 297 affected relatives. Overall patient characteristics are summarized in Table 2.

**Table 2:** Comparison of clinical profile and features of unrelated Thai patients with SCA1, SCA2, MJD and SCA6

	**SCA1**	**SCA2**	**MJD**	**SCA6**	*p*-value*
Total numbers	21	15	39	8	
Sex (M/F)	11/10	8/7	14/25	4/4	0.500
Age at onset (years)					
Mean age at onset	38.43	41.47	39.97	43.5	0.715
SD	10.14	13.01	12.17	6.76	
Range	15 – 55	19 - 60	16 - 64	34 - 58	
Duration (years)					
Mean duration	5.43	3.93	6.62	6	0.328
SD	5.13	2.99	5.01	5.90	
Range	1 - 20	1 - 10	0.25 - 22	1 - 18	
Positive family history (%)	16 (76.2)	13 (86.7)	32 (82.1)	7 (87.5)	0.900
CAG repeat size (repeats)					
Mean	50.14	37.40	69.97	21.88	
SD	6.27	4.47	4.04	0.83	
Range	41 – 65	32 - 52	62 - 78	21 - 23	
Clinical features (%)					
Slow saccade	12 (57.1)	7 (46.7)	21 (53.9)	2 (25)	0.454
Horizontal nystagmus	5 (23.8)	4 (26.7)	34 (87.2)	6 (75)	0.0000002**
Vertical nystagmus	0 (0)	1 (6.7)	6 (15.4)	3 (37.5)	0.026**
Opthalmoparesis	6 (28.6)	3 (20.0)	26 (66.7)	2 (25)	0.002**
Pale optic disc	1 (4.8)	0 (0)	2 (5.1)	1 (12.5)	0.610
Hyperreflexia	19 (90.5)	5 (33.3)	26 (66.7)	7 (87.5)	0.002**
Babinski’s sign	11 (52.4)	5 (33.3)	17 (43.6)	5 (62.5)	0.510
Areflexia	0 (0)	5 (35.7)	9 (23.1)	0 (0)	0.011**
Sensory impairment	4 (19.0)	3 (20.0)	9 (23.1)	0 (0)	0.630
Parkinsonism	0 (0)	0 (0)	0 (0)	0 (0)	1.000
Dystonia	1 (4.8)	0 (0)	1 (2.6)	0 (0)	0.475
Chorea	0 (0)	0 (0)	1 (2.6)	0 (0)	1.000
Dementia	0 (0)	1 (6.7)	0 (0)	1 (12.5)	0.074
Facial fasciculation	2 (9.5)	0 (0)	5 (12.8)	0 (0)	0.579
SARA					
Mean scale	16.97	13.18	16.76	15.71	0.767
SD	7.45	3.66	7.27	7.24	
Range	4 - 30	9 - 20	8 - 35	3.5 - 27	

*Continuous variables were analyzed by using ANOVA. Comparisons of frequencies of clinical profiles and features were analyzed by using Fisher’s exact test.

**P < 0.05 is considered as significant difference.

*Abbreviations as follows:SCA* spinocerebellar ataxia, *MJD* Machado-Joseph disease, *SD* standard deviation, *SARA* scale for the assessment and rating of ataxia.

**Figure 1:**
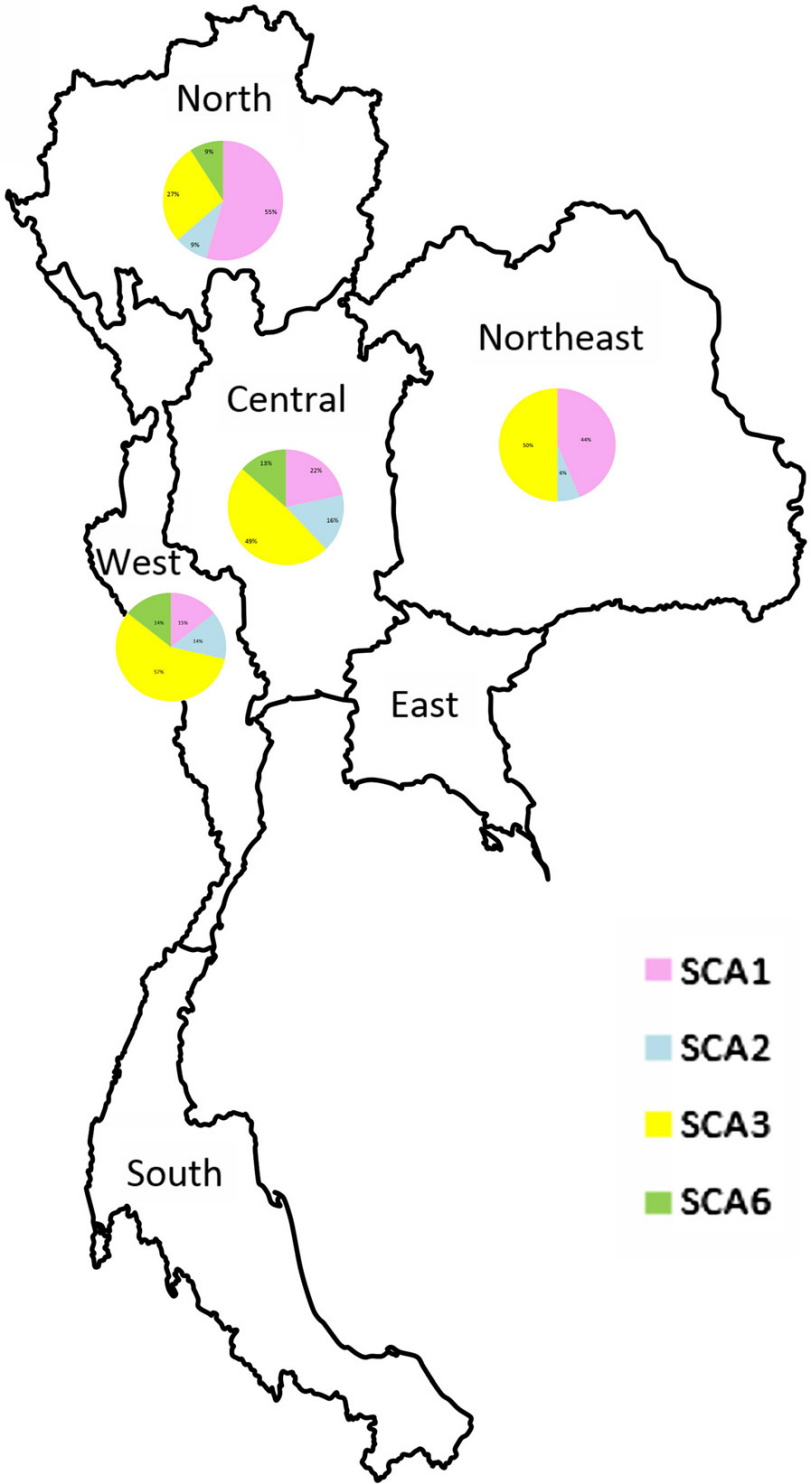
Map of Thailand illustrates the distribution of the frequencies of the common SCAs.

Thailand is geographically divided into six regions comprising central, north, northeast, east, west and south. Most of the patients in the study lived in central, north, west and northeast of Thailand. Only four patients were referred from eastern (2) and southern (2) regions (Figure 1). In central, western and northern regions, we identified patients with SCA1, SCA2, MJD and SCA6. However, almost all families living in the northeastern region were SCA1 and MJD. Only one family was SCA2 and no SCA6 family was identified in the region.

General factors including gender, age at onset, duration of disease, positive family history and SARA scale were not different among the different types of SCAs (Table 2). Average age at onset was around late thirties to early forties in all groups. SCA6 did not correlate with later age at onset compared to the other SCAs (43.5±6.76, mean±SD). Furthermore, none of the studied SCA6 patients had age at onset after 60 years. Durations of the disease and SARA scales were similar in all groups.

### Clinical features

Gait ataxia or unsteadiness was the presenting symptom in all cases. Arm incoordination and cerebellar dysarthria generally followed within a few to several years. Limb ataxia was sometimes asymmetrical; however difference of severity was frequently not in a large degree. Truncal ataxia was uncommon until the advance stage of the disease. In the early stage, neurological signs including dysmetric saccades, nystagmus and pyramidal signs particularly increased tendon reflexes and spastic tone in both legs could often be observed. We had the opportunity to examine several asymptomatic carriers of MJD at the age between 22 to 30 years. Some of them had brisk tendon reflexes and mild spasticity in both legs, although they did not have gait unsteadiness.

Frequencies of nystagmus, upward gaze paresis, hyperreflexia and areflexia were significantly different among different subtypes of SCAs (Table 1). Nystagmus was commonly gaze-evoked type. Horizontal nystagmus was more common than vertical nystagmus. It was relatively more common in MJD and SCA6 than the other types. Ophthalmoparesis was invariably upward direction and it was significantly more frequent in MJD than other SCAs. Almost all patients who exhibited upward gaze paresis, were nuclear type. Supranuclear gaze paresis was rare in the patients studied and it was observed in only a few patients in the early stage of the disease. Severe ophthalmoparesis in all directions was observed in only a few patients with advance stage of SCA1 and MJD.

Increased tone and tendon reflexes were generally more prominent in the lower extremities. It was common in all studied subtypes of SCAs except SCA2. It was noted that almost all patients with SCA1 (90%) developed pyramidal signs. One of the only two SCA1 patients, who did not have pyramidal signs, had severe generalized dystonia. Areflexia was often observed in only ankle reflexes and it was less often accompanied with absent knee reflexes or generalized areflexia. Absent ankle reflexes were also observed in the presence of brisk knee reflexes and Babinski’s sign. Areflexia was not apparent in both SCA1 and SCA6 in the study. Sensory symptoms were usually subtle and the impairment of vibration or pin prick sensations was observed in about one-fifth of the SCA1, SCA2 and MJD patients. Only two patients studied (SCA2 and MJD) had severe axonal sensorimotor polyneuropathy. They developed generalised areflexia, marked distal wasting, mild distal weakness and sensory loss in gloves and stockings pattern since the early stage of the disease.

Abnormal saccadic eye movements were demonstrated in all subtypes of the studied SCAs. Slow saccades were found in about half of the patients. With the exception of SCA6, it was observed in only a quarter of the patients. However, frequencies of slow saccades were not different among different SCAs (p = 0.454). Dysmetric saccades (both hypermetric and hypometric saccades) and impairment of vestibulo-ocular-reflex suppression were also identified in a large proportion of the examined patients of all SCA subtypes. However, we did not systematically record this data from the beginning of the study.

Facial fasciculation, dystonia, chorea and cognitive impairment were uncommon in the patients studied. Facial fasciculation was only observed in a few patients with SCA1 and MJD. Both periorbital and orolingual distribution was equally observed, and it was often asymmetry. Fasciculation was much rarer in the extremities. Dystonia was actually observed in only two families with SCA1 and MJD. The SCA1 patient had severe generalized dystonia and the MJD patient had unilateral foot dystonia prominently only while walking. None of the patients studied had signs of parkinsonism. Generalized chorea was present in two unrelated MJD patients including one index case and one affected family members (not included in the analysis). Both patients were in the advanced stage. One of the patients had been wheel-chair bound and the other had been confined to bed for over 3 years.

### Disease severity and non-ataxic symptoms

Comparisons of demographic data and clinical features between patients with SARA ≥ 15 and <15 are shown in Table 3. Since age at onset and age are correlated, they theoretically may cause multicollinearity. Therefore, we applied two separate models of logistic regression analyzes by using age at onset or age as parameter. After performing multivariate analysis, ophthalmoparesis and sensory impairment were shown to be independently associated with disease severity by both models (Table 4). Age was also a factor associated with severity of the disease when age at onset was excluded from the analysis (model 2 in Table 4).

**Table 3:** Comparison of baseline factors between SARA ≥15 and SARA <15 groups

**Factors**	**SARA ≥15 N = 49 (%)**	**SARA <15 N = 34 (%)**	***p*-value**
Sex			
Female	23 (46.9)	14 (41.2)	0.603
Age onset (years); mean (SD)	42.1 (11.1)	37.5 (11.3)	0.071
Age when exam (years); mean (SD)	50.5 (11.7)	39.5 (11.3)	<0.001
Duration (years); mean (SD)	8.9 (5.1)	3.4 (2.2)	<0.0001
SCA Type			
SCA 1	13 (26.5)	8 (23.5)	0.325
SCA 2	6 (12.2)	9 (26.5)	
SCA 3	26 (53.1)	13 (38.2)	
SCA 6	4 (8.2)	4 (11.8)	
Family history	44 (89.8)	24 (70.6)	0.025
Slow saccades	29 (59.2)	14 (41.2)	0.106
Horizontal nystagmus	32 (65.3)	17 (50)	0.163
Vertical nystagmus	8 (16.3)	2 (5.9)	0.187
Upward gaze paresis	31 (63.3)	6 (17.6)	<0.001
Hyperreflexia	37 (75.5)	20 (58.8)	0.107
Areflexia	10 (20.4)	4 (11.8)	0.301
Babinski’s sign	26 (53.1)	12 (35.3)	0.110
Sensory impairment	14 (28.6)	2 (5.9)	0.010
Optic atrophy	4 (8.2)	0 (0)	0.141

**Table 4:** Univariate and multivariate logistic regression analyses of parameters associated with more severe ataxia group (SARA ≥ 15); (Tables show only significant parameters)

**Univariate analysis**				
**Variable**	**All 4 common SCAs**		**MJD, SCA1 and SCA2**	
	**Odd ratio (95% CI)**	***p*-value**	**Odd ratio (95% CI)**	***p*-value**
Age at onset (years)	1.04 (0.99-1.08)	0.075	1.04 (1.01-1.09)	0.036
Age when assessment (years)	1.08 (1.04-1.13)	<0.001	1.08 (1.04-1.14)	<0.001
Positive family history	3.67 (1.12-11.97)	0.031	5.13 (1.43-18.37)	0.012
Ophthalmoparesis	8.07 (2.80-23.10)	<0.001	7.25 (2.45-21.41)	<0.001
Sensory impairment	6.40 (1.35-30.37)	0.019	6.32 (1.32-30.31)	0.021
Babinski’s sign	not significant		2.67 (1.01-7.07)	0.049
**Multivariate logistic regression analysis: model 1** Consider age at onset as parameter				
**Variable**	**All 4 common SCAs**		**MJD, SCA1 and SCA2**	
	**Odd ratio (95% CI)**	***p*-value**	**Odd ratio (95% CI)**	***p*-value**
Ophthalmoparesis	8.19 (2.74-24.44)	<0.001	7.56 (2.44-23.42)	<0.001
Sensory impairment	6.64 (1.27-34.71)	0.025	6.82 (1.29-36.01)	0.024
**Model 2** Consider age as parameter				
**Variable**	**All 4 common SCAs**		**MJD, SCA1 and SCA2**	
	**Odd ratio (95% CI)**	***p*-value**	**Odd ratio (95% CI)**	***cx*-value**
Age when assessed	1.07 (1.02-1.13)	0.004	1.08 (1.03-1.14)	0.003
Ophthalmoparesis	6.18 (1.90-20.03)	0.002	5.62 (1.64-19.26)	0.006
Sensory impairment	9.28 (1.43-60.07)	0.019	9.41 (1.40-63.34)	0.021

### Sizes of expanded SCA3 alleles and non-ataxic symptoms

Univariate logistic regression analysis revealed that size of the pathological SCA3 repeat allele >70 was associated with the presence of increased tendon reflexes (OR = 5.33, 95% CI = 1.17-25.21, p = 0.03). However, the association was excluded after adjustment with age, age at onset, sex and disease duration (OR = 2.38, 95% CI = 0.56-10.03, p = 0.24).

## Discussion

Overall, MJD was the most common SCA in Thailand similar to other East Asian countries except Korea, in which SCA2 appeared to be the most common SCA [30]. DRPLA is rare in Chinese and Thais, while it is relatively common in Japan, Singapore and Korea [30-32]. Apart from MJD, SCA1 and SCA2 are also prevalent in most East Asian populations, in contrast to Japanese, in which SCA6 and SCA31 are the second common SCA, and frequencies of SCA1 and SCA2 are less common [31,33]. We described clinical characteristics of the four most common SCAs in Thailand including MJD, SCA1, SCA2 and SCA6. This data showed that the main clinical features of Thai patients were similar to the previous reports of other ethnic groups to some extent [9,13,15,16,34-37]. However, there were also noticeable differences in some aspects between Thai patients and patients of other ethnic groups including (1) frequent observation of slow saccades in all of the studied types of SCA; (2) the relatively less frequent neuropathy and more frequent pyramidal signs in SCA2; (3) the relatively earlier onset of SCA6 in Thais; and (4) the scarcity of extrapyramidal features and cognitive impairment. Nevertheless, phenotypes of the common SCAs are generally very heterogeneous and frequencies of associated non-ataxic symptoms among different populations are widely diverse. Therefore, the findings are not surprising. However, these data suggest that the clinical clues for differential diagnosis of the common types of SCAs such as age at onset, slow saccade and areflexia may be less useful in the Thai patients.

Slow saccade has been well-known as one of the major non-ataxic features of SCA2 [9,17,22,38]. Reduced saccadic velocity can be apparent in presymptomatic carriers of SCA2 implying that oculomotor pontine nuclei likely to be susceptible to neurodegenerative process similar to cerebellar neurons [19]. Previous evidence also suggested that the occurrence of slow saccade might be associated with size of the pathological repeat alleles and its severity was subsequently progressive over time [17,22]. Slow saccade was less often reported in association with SCA1, MJD and SCA6 [39], though our data showed similar frequencies of slow saccade among these SCAs. So the mechanism underlying the susceptibility of specific neurons other than cerebellar neurons to the neurodegenerative process caused by these polyglutamine diseases is likely to be complex and may involve other genetic factors apart from specific genes and the size of the pathological repeat alleles.

Not only is the frequency of peripheral neuropathy often more common in SCA2 than the other common subtypes, the SCA2 patients also develop polyneuropathy at an early stage of the disease [40]. A large European study revealed that the occurrence of peripheral neuropathy in SCA2 was not associated with disease duration. In contrast to MJD and SCA6, polyneuropathy might relate to disease duration, which tended to develop in the later stage [15]. These data implied that a susceptibility to neuropathy was greater associated with the trinucleotide repeat expansion in *ATXN2* gene than the others. Although polyneuropathy was less frequent in the Thai SCA2 patients compared to other ethnic groups, and frequencies of polyneuropathy were not much different between SCA2 and MJD. The study also observed that evidence of peripheral neuropathy appeared to be occurrence in the early stage of SCA2 in contrast to a later stage in association with MJD similar to previous reports.

SCA6 has been generally known to be progressive ataxia with later onset compared to SCA1, SCA2 and MJD, in which age at onset may be after 60 years old [41-43]. Some evidence suggested that age at onset of SCA6 might be inversely related to size of CAG repeats, with the larger size of the CAG repeats more likely to be associated with the early age at onset [42]. All of the patients studied had sizes of CAG repeats of 23 or lower, thus it is unlikely that this finding related to the size of CAG repeat. However, the study group is small. So we then further reviewed medical records of all of the ten SCA6 families in our clinics. Of the 18 patients from unrelated 10 SCA6 families, mean age of onset was 42.6 years, range 34–61 years. Only one patient had age at onset over 60 years old. Regarding the data of age at onset of the Thai SCA6 patients together with a high frequency of prominent pyramidal signs, nystagmus and saccadic abnormalities, it is difficult to clinically distinguish SCA6 from MJD, SCA1 and SCA2.

In the study, the Thai SCA patients rarely exhibit parkinsonism, dystonia, dementia and facial fasciculation. Parkinsonism has been reported to be relatively common in SCA2 and SCA3 [44-47]. None of the studied patients exhibited parkinsonian features. In the study, we evaluated cognitive functions by using history taking and bedside examination. Almost all of the patients did not clinically have cognitive impairment and dementia. It was quite uncommon even in the late stage of the disease. Regarding dystonia, it has not been a commonly described non-ataxic feature of SCAs [48], dystonia is also extremely rare in the Thai patients.

The multiple regression analyses suggested that the development of ophthalmoparesis and polyneuropathy may be associated with severity of the disease and age of the patients rather than the subtypes of SCAs. However, the study had some limitations. The sample size is relatively small since we recruited only index cases of each family. So the study performed two logistic regression analyses including: (1) all patients together, and (2) all patients except SCA6. Since underlying pathological process of SCA6 is a small CAG expansion, which is different from other SCAs studied. Secondly, a large proportion of our patients were of low socioeconomic and low-educated status. They might give inaccurate data of some important information such as age at onset and disease duration.

## Conclusions

In summary, MJD, SCA1, SCA2 and SCA6 are common in Thai adult-onset cerebellar degeneration, in contrast to SCA7 and DRPLA which are rare. The Thai patients with MJD, SCA1, SCA2 and SCA6 often exhibited a similar non-ataxic phenotype including pyramidal features prominently in lower limbs, saccadic abnormalities and less often peripheral neuropathy. The study shows that slow saccades and age onset are of no value for differential diagnosis of the common SCA subtypes in Thailand. Thus, it is essential to set up an effective referral system for reachable genetic diagnosis of a panel of at least the four common SCAs in Thailand.

## Abbreviations

SCA: Spinocerebellar ataxia; MJD: Machado-Joseph disease; DRPLA: Dentatorubro-pallidoluysian atrophy; SARA: Scale for the assessment and rating of ataxia; ANOVA: One-way analysis of variance; OR: Odd ratio; 95% CI: 95% confidence interval.

## Competing interests

All authors declare no competing interests.

## Authors’ contributions

PB, study design, clinical analyses. SP, review medical records, recruit cases and data collection. PP, study design, collect data, analysis of pilot data. CP, genetic analysis. SW, recruit cases and data collection. SP, recruit cases and data collection. ST, recruit cases and data collection. AN, recruit cases and data collection. CD, study design, data collection. PJ, clinical analyses especially on examination of eye movements. CJ, clinical analyses, data collection. DM, statistical analysis. AI, statistical analysis. TP, principal investigator, study design, clinical and statistical analysis, writing manuscript. All authors read and approved the final manuscript.

## Pre-publication history

The pre-publication history for this paper can be accessed here:

http://www.biomedcentral.com/1471-2377/14/75/prepub
